# HER2 overexpression a major risk factor for recurrence in pT1a-bN0M0 breast cancer: results from a French regional cohort

**DOI:** 10.1002/cam4.167

**Published:** 2014-01-10

**Authors:** Philippe Rouanet, Pascal Roger, Emilie Rousseau, Severine Thibault, Gilles Romieu, Andre Mathieu, Jacques Cretin, Gilbert Barneon, Mireille Granier, Aurelie Maran-Gonzalez, Jean P Daures, Florence Boissiere, Frederic Bibeau

**Affiliations:** 1 Montpellier Cancer Institute/Val d'AurelleMontpellier, France; 2 Department of pathology, CHU NimesNimes, France; 3 Biostatistics, epidemiology and clinical research unit, IURCMontpellier, France; 4 Onco LRMontpellier, France; 5 Department of pathology, CHU MontpellierMontpellier, France

**Keywords:** HER-2 positive tumors, small breast carcinoma

## Abstract

The management of pT1a-bN0M0 breast cancer remains an area of controversy. Data from 714 patients classified as having pT1a-bN0M0 breast cancer and treated, from 1999 to 2004 in the Languedoc-Roussillon France, were analyzed. The human epidermal growth factor receptor 2 (HER2) status analyses were centralized. The objective of this study was to describe the prognosis of pT1a-bN0M0 breast cancer according to HER2 distribution and hormonal status. The median follow-up was 6.4 years. Ten-year overall survival was 94%. HER2 overexpression was observed in 6.1% of the patients. The 10-year prognosis of patients with HER2-positive tumors was worse than that of those with HER2-negative (disease-free survival 73% vs. 89%, *P* < 0.0001). Tumor size (T1a/T1b) was not a relevant prognostic factor. The co-expression of HER2 with hormonal receptors (HR) was associated with high recurrence at 10 years. In both univariate and multivariate analyses, the most relevant prognostic factor for this population was HER2 amplification. In multivariate analysis, patients with HER2-positive tumors had higher risk of mortality (HR, 3.89; 95% CI, 1.58–9.56). In pT1a-bN0M0 breast cancers, HER2 amplification or overexpression is a risk factor for recurrence. In HER2-positive breast cancers, HR expression is associated with a poor prognosis despite the hormone therapy. For this population, a personalized management may be required.

## Introduction

The diagnosis of small breast carcinoma (SBC), defined as pT1a-bN0M0, is largely increasing with the introduction of mammographic screening and prevention programs. Although the prognosis of patients with pT1a-bN0M0 breast cancer is generally good, it is still unclear how to personalize adjuvant treatment according to subgroups.

Published data concerning the clinical outcome of patients with SBC and receiving local therapy only, reported 10 and 20-year disease-free survival (DFS) rates of more than 90% and 80%, respectively 1,2. However, 6–12% of these SBCs overexpressing the human epidermal growth factor receptor 2 (HER2-positive) 3, are correlated with poorer prognosis including higher risk of recurrence and mortality. The 5-year DFS in patients with HER2-positive SBC treated with local therapy only ranged between 77% and 95% 4.

The use of adjuvant systemic therapy has led to substantial improvements in prognosis for patients with breast cancer, but it remains under debate for this subset of SBC 5,6.

Trastuzumab, a monoclonal antibody targeted against HER2, in combination of chemotherapy is currently the gold standard for patients with HER2-positive tumors larger than 1 cm. Patients with stage T1a-bN0 disease were excluded from the large randomized controlled trials that established the efficacy of trastuzumab in early-stage breast cancer 7–10. In the absence of clinical evidence, the use of trastuzumab-based therapy remains controversial considering the slight risk of cardiac dysfunction reported in these trials. The decision to use anti-HER2 based therapies must be individualized, taking into account known toxicities, patient preferences, and the uncertain benefits 4.

Current National Comprehensive Cancer Network (NCCN) guidelines version 3.2012 do not recommend adjuvant chemotherapy or anti-HER2 therapy for T1aN0 tumors, and have only suggested considering these systemic therapies for patients with HER2-positive T1bN0 disease and additional unfavorable features, such as hormone receptor-negative (HR-negative) tumor, extensive lymphovascular space invasion, or moderately/poorly differentiated tumors.

In this article, we describe a large cohort of patients with exhaustive regional recruitment, long follow-up period, and centralized HER2 screening. The objective of the current analysis was to determine the prognostic factors for recurrence in patients diagnosed with T1a-b and lymph node-negative breast carcinoma, stressing on HR and HER2 status.

## Patients and Methods

### Study population

All the patients diagnosed with pT1a-b pN0 breast cancer and treated in the Languedoc-Roussillon Region in France within the Onco LR network (Southern French regional network of cancer institutions) from 1999 to 2004, were selected for this study. A chart review via the Onco LR database (based in the University Institute of Clinical Research—Montpellier I) was conducted to identify the patients and the baseline characteristics. The data concerning the follow-up were then extracted from the patients' medical records.

**Figure 1:**
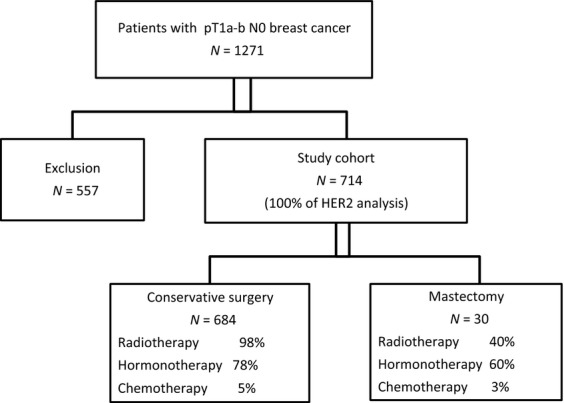
Flow chart of study population.

We identified 1217 women diagnosed with pT1a-bpN0M0 breast cancer between 1999 and 2004 from the Onco LR database (Fig. 1). Of these, 557 were excluded for the following reasons: 226 had insufficient material, 98 tumors were multifocal, 67 had positive margins (invasive or ductal in situ carcinoma) without certainty for a conservative curative resection, 59 had previous breast carcinoma, 56 had received neoadjuvant treatment, 37 tumors were bilateral and 14 had ductal carcinoma in situ (DCIS) associated with microinvasion. Study population consisted of the remaining 714 unilateral pT1a-b pN0 breast cancers.

The population included patients who had undergone either mastectomy or breast-conservation therapy (BCT). For breast-conservation patients, the standard radiotherapy approach during the study period was adjuvant whole breast radiation therapy consisting of 50 Gy in 25 fractions followed sometimes by a boost of 10 Gy in five fractions.

### Immunohistochemistry

Immunohistochemistry (IHC) was performed to determine the HR status following the standard procedures using 4-*μ*m sections of paraffin-embedded tissues marked with monoclonal antibodies for HR. The cutoff for positive expression of either estrogen receptor (ER) or progesterone receptor (PR) was defined as 10% or more of cells stained. Tumor grading was assessed as defined by Elston and Ellis.

**Figure 2:**
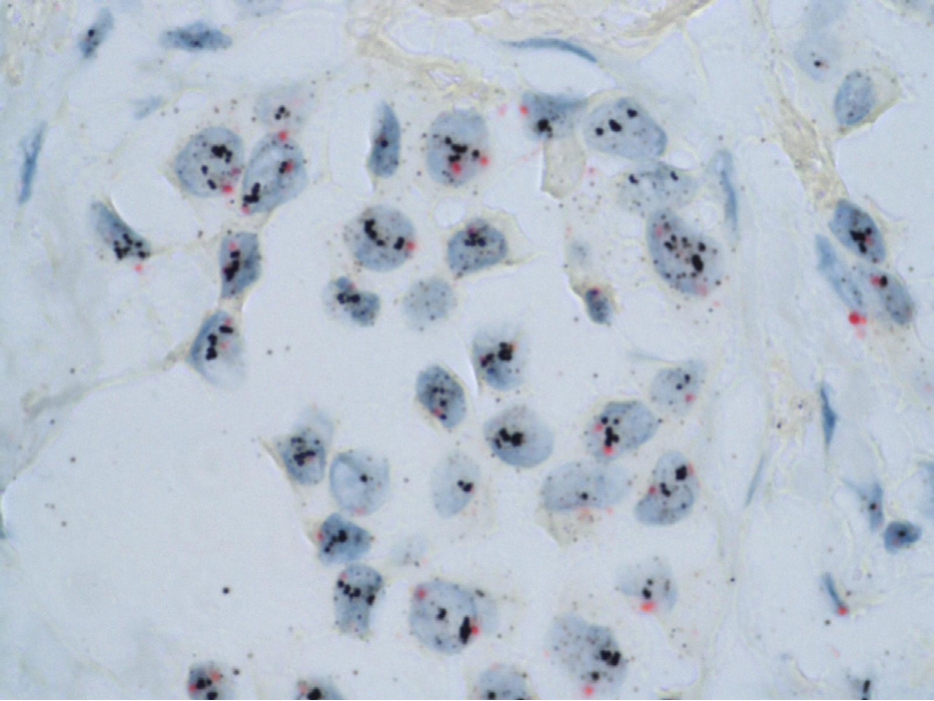
Determination of *HER2* gene status using the Dual SISH kit (Ventana) of a breast carcinoma with *HER2* gene amplification; HER2 (black) and Chr17 (red) 100×. HER2, human epidermal growth factor receptor 2; SISH, silver in situ hybridization.

For all patients in the study, HER2 status was analyzed centrally by IHC using the 4B5 monoclonal antibody (Ventana Medical Systems Inc., Tucson, AZ) on the BenchMark XT system (Ventana Medical Systems Inc.). Invasive breast carcinoma (BC) were graded according to the American Society of Clinical Oncology/College of American Pathologists (ASCO/CAP) guideline recommendations. Cases showing no membrane immunostaining or in less than 10% invasive cancer cells were scored 0+; cases with weak and incomplete membrane staining in more than 10% of invasive cancer cells were scored 1+; cases with complete weak or moderate membrane staining in at least 10% of cells were scored 2+ and cases with strong membrane staining in more than 30% invasive tumor cells were scored 3+. In each case, benign glands were used as internal negative controls for specificity and an invasive breast cancer case with known HER2 overexpression was used as an external positive control. Cases were assessed by experienced pathologists blinded to patient information. All equivocal cases were reviewed in three dedicated sessions for final consensus. All the tumors with score 2+ and equivocal IHC results were tested for gene amplification by Dual color SISH (silver in situ hybridization) (Fig. 2).

SISH is an alternative technique to FISH (fluorescent in situ hybridization) to determine whether breast carcinoma cells dysplay an HER2 amplification or not. The major advantage of SISH is explained by an easy use in pathology laboratories, which does not require a specific environment for fluorescent techniques. Thus, SISH belongs to the so-called bright-field techniques, which only use light microscopy and no fluorescent microscopy. In other terms, it is a more accessible tool for pathologists, especially in daily routine. Furthermore, SISH provides a very good resolution, combining an excellent preservation of tumor morphology and a precise definition of amplified cells with silver coloration. DualSISH allows the visualization of both HER2 and centromere loci with spectific probes. Dual color SISH analyses were carried out using an automated system (BenchMark XT system) following the manufacturer's protocols for INFORM HER2 DNA and chromosome 17 probes 11. Both probes were sequentially hybridized on the same slide. HER2 gene and chromosome 17 single copies were visualized as black dots and as red dots, respectively. Results were interpreted according to the ASCO/CAP guidelines 12. HER2 and CEP17 signals were enumerated in at least 30 nuclei within a target area and the HER2/CEP17 ratio was calculated. Cases with a HER2/CEP17 ratio <1.8 were considered negative for HER2 amplification and those with a HER2/CEP17 ratio >2.2 were considered positive for HER2 amplification. If a HER2/CEP17 ratio fell on or between the values of 1.8 and 2.2, the number of signals in additional 20 nuclei was counted in a second target area. The HER2/CEP17 ratio was then calculated from both target areas (at least 60 cells). HER2-positive BCs were defined as IHC overexpressed (score 3+) or amplified.

IHC and dual-SISH evaluations allowed us to classifying patients into four distinct BC subtypes: HER2+HR+, HER2+HR−, HER2−HR+ and, HER2−HR−.

### Statistical analyses

Patients were categorized according to HER2 status. Patient characteristics were tabulated by median and range, and were compared between groups with the chi-square test or Wilcoxon rank sum test, as appropriate. Time to recurrence was measured from the date of diagnosis to the date of first local or distant disease recurrence, or to the last follow-up. Patients who died before experiencing a disease recurrence were considered censored at the dates of death. Time to recurrence and time to distant recurrence were estimated according to the Kaplan–Meier method and were compared between groups with the log-rank statistic. Cox proportional hazards models were fit to determine the association of HER2 status with the risk of recurrence after adjustment for other patient and disease characteristics. *P*-values <0.05 were considered statistically significant. The statistical software package SAS version 9.3 (SAS Institute, Cary, NC) was used for statistical analyses.

## Results

### Patient characteristics

The median age at diagnosis was 59 years (range 32–84); 20% of women were less than 50 years old. Of the 714 patients, 133 were pT1a (19%) and 581 were pT1b (81%).

The histological type was mostly ductal (75% vs. 15% lobular). Nuclear grade was I in 47% of tumors, II in 48%, and only 5% were grade III.

The majority of patients (91%) had HR-positive tumor, with 87% ER-positive tumor. Tumors were HER2-positive in 6.1% of patients (*n* = 44); of these 3.6% (*n* = 26) were HR-positive. The remaining patients (7%, *n* = 47) had triple negative (TN) breast cancer.

Most of patients (96%, *n* = 684) had undergone BCT and 4% (*n* = 30) mastectomy. Indication for mastectomy was always associated to an extensive intraductal component. Of those who underwent mastectomy, 12/30 received postoperative radiotherapy. Hormonal therapy was administered in 554 patients (78%), anti-estrogen therapy in 91% and aromatase inhibitors in 8%. Few patients (5%, *n* = 36) received chemotherapy. None received adjuvant anti-HER2 therapy.

### Ten-year outcomes for patients with pT1a-b N0 breast cancer

With a median follow-up of 6.4 years (range 0.03–9.99), a total of 38 recurrences occurred (5%), including 15 contralateral tumors, 12 isolated distant recurrences, 10 isolated loco-regional recurrences (six in the breast, four nodal), and 1 local and distant recurrence. Twenty-nine patients died: 16 unrelated with the tumor, seven due to the tumor and six for an unknown reason.

The 10-year overall survival (OS) and DFS was 94% (95% CI 90–96%) and 88% (95% CI 84–91%), respectively. The local recurrence-free survival (LRFS) was 97% and the metastasis-free survival (MFS) was 88%.

Table 1 resumes the impact of clinical characteristics on the prognosis. The age and the HER2/HR status were significantly influencing the survival. Size of the tumor and histology had no significant impact.

**Table 1:** Ten-year outcomes in 714 patients with pT1a-bN0M0 breast cancer and main prognostic factors.

		10-year outcomes											
		OS			DFS			LRFS			MFS		
Variables	No.	*N*	%	*P*^1^	*N*	%	*P*^1^	*N*	%	*P*^1^	*N*	%	*P*^1^
All	714	29	94		59	88		11	97		39	88	
Age													
≤50	155	1	98	0.015	9	92	0.200	5	96	0.063	3	96	0.031
>50	559	28	92		50	87		6	98		36	87	
Tumor size													
1–5 mm	133	5	94	0.781	14	86	0.367	4	96	0.153	6	94	0.580
6–10 mm	581	24	93		45	89		7	98		33	88	
Histology													
Ductal	530	22	94	0.235	45	89	0.344	9	97	0.736	30	87	0.310
Lobular	106	6	86		10	79		1	96		7	83	
Other	78	1	98		4	94		1	98		2	97	
Nuclear grade													
I	332	9	96	0.050	24	89	0.051	4	98	0.420	15	93	0.017
II	335	16	91		28	87		7	96		18	84	
III	36	4	87		7	80		0	–		6	83	
HR status													
Positive	649	23	94	0.046	50	88	0.113	9	98	0.316	33	88	0.204
Negative	65	6	88		9	83		2	95		6	89	
HER2 status													
Positive	44	6	84	0.001	11	73	<0.0001	3	92	0.003	8	80	<0.0001
Negative	670	23	94		48	89		8	98		31	89	

OS, overall survival; DFS, disease-free survival; LRFS, local recurrence-free survival; MFS, metastasis-free survival; HR, hormonal receptors; HER2, human epidermal growth factor receptor 2.

1 Log rank test was performed to compare between subgroups.

### Characteristics and outcomes according to HER2 status

Main patient and tumor characteristics according to HER2 status are presented in Table 2. Compared with HER2-negative tumors, HER2-positive were more frequently T1a (*P* < 0.0001), less frequently HR-positive (*P* < 0.0001), and had higher Scarff Bloum and Richardson grades (*P* < 0.0001).

**Table 2:** Clinical and pathologic characteristics of patients with pT1a-bN0M0 breast cancer according to HER2 status.

	HER2-negative (*N *=* *670)		HER2-positive (*N *=* *44)		
Characteristics	*N*	%	*N*	%	*P-*value
Age at diagnosis, years					
≤50	147	22	8	18	0.5580
>50	523	78	36	82	
Tumor size					
1–5 mm	114	17	19	43	<0.0001
6–10 mm	556	83	25	57	
Histological type					
Ductal	491	73	39	89	0.0617
Lobular	102	15	4	9	
Other	77	11	1	2	
SBR grade					
I	328	50	4	9	<0.0001
II	305	46	30	68	
III	26	4	10	23	
ER status					
Positive	601	90	22	50	<0.0001
Negative	69	10	22	50	
PR status					
Positive	473	71	21	48	0.0015
Negative	197	29	23	52	
HR status					
Positive	623	93	26	59	<0.0001
Negative	47	7	18	41	
Initial local treatment					
Mastectomy	22	3	8	18	<0.0001
BCS + Radiotherapy	638	95	34	77	
BCS alone	10	2	2	5	
Initial systemic treatment					
None	116	17	24	55	<0.0001
Hormonotherapy alone	522	78	16	35	
Chemotherapy alone	18	3	2	5	
Hormonotherapy + chemotherapy	14	2	2	5	

HER2, human epidermal growth factor receptor 2; ER, estrogen receptor; PR, progesterone receptor; HR, hormonal receptors; SBR, Scarff Bloum and Richardson; BCS, breast conservative surgery.

HER2-positive tumors were less often treated with a BCT and radiotherapy (77% vs. 95%) and the majority did not receive systemic treatment (54%). When systemic treatment was administered, hormone therapy was more frequent (36%) than chemotherapy (5%). Among the 44 patients with HER2-positive BC, 26 patients (60%) had HR-positive tumors and 18 patients (40%) had HR-negative tumors.

**Figure 3:**
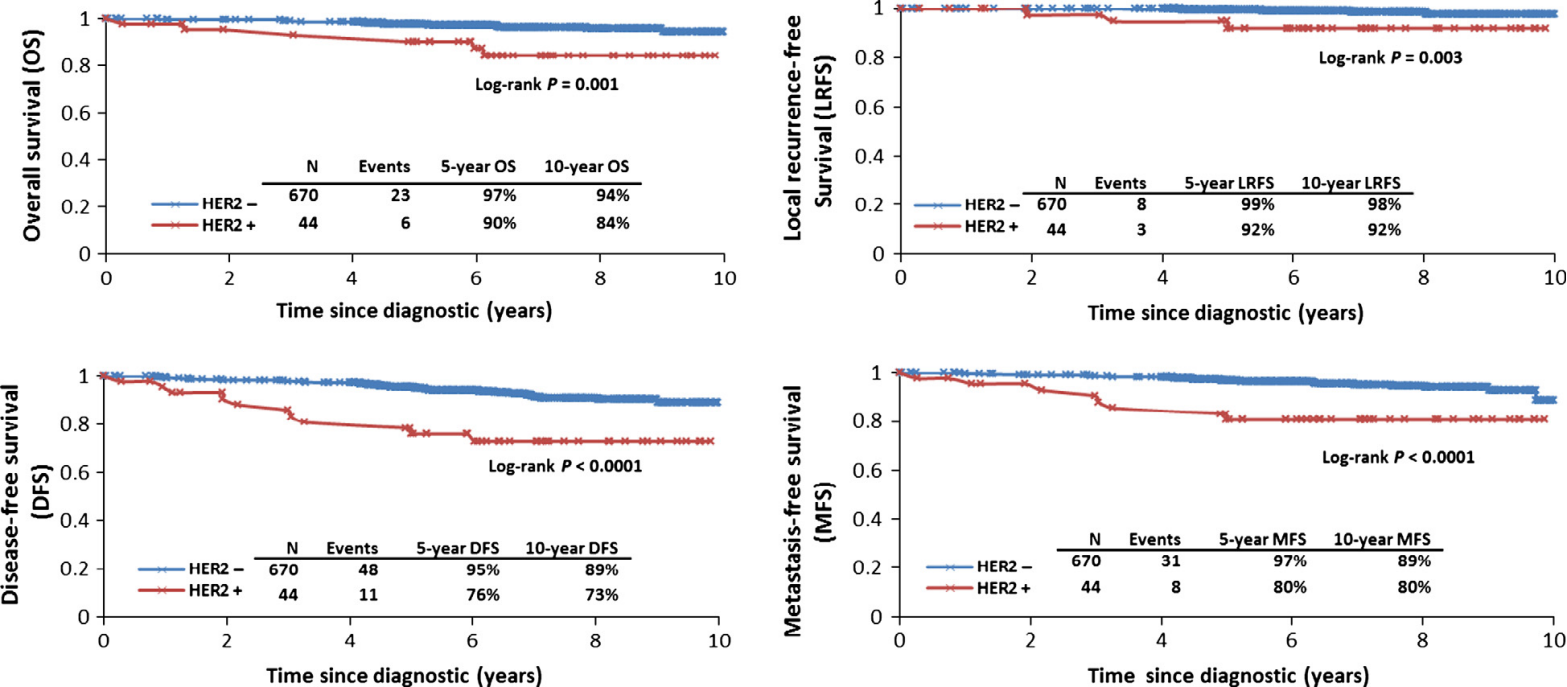
Ten-year prognosis in patients with pT1a-bN0M0 breast cancer according to human epidermal growth factor receptor 2 (HER2) status.

At 10-year follow-up, patients with HER2-positive breast cancer had a worse prognosis than those with HER2-negative breast cancer. Figure 3 shows the impact of HER2-positivity on OS (84% vs. 94%; *P* = 0.001), DFS (73% vs. 89%; *P* < 0.0001), LRFS (92% vs. 98%; *P* = 0.003) and MFS (80% vs. 89%; *P* < 0.0001).

When considering groups according to HER2 and HR status, the distribution of patients was as follows: HER2−HR+ 87% (*n* = 623); HER2−HR− 7% (*n* = 47); HER2+HR+ 4% (*n* = 26) and HER2+HR− 2% (*n* = 18).

**Figure 4:**
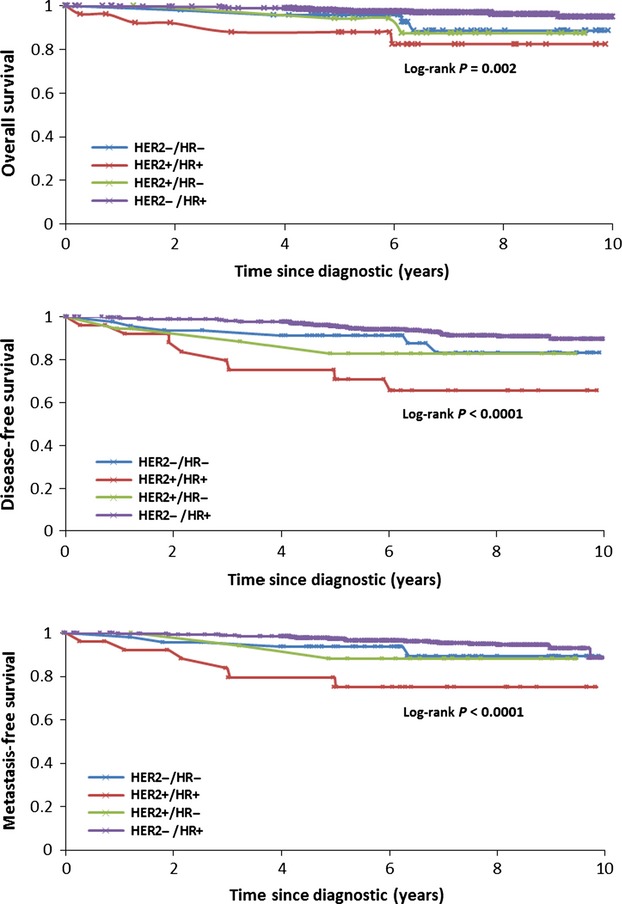
Ten-year prognosis in patients with pT1a-bN0M0 breast cancer according to HR/HER2 status. HR, hormonal receptor; HER2, human epidermal growth factor receptor 2.

The clinical outcomes according to HR and HER2 status are presented in Figure 4. HER2−HR+ tumors had the better 10-year prognosis, including an OS of 94% [90–97%] and DFS of 89% [85–93%]. The prognosis of TN tumors (HER2−HR−) was also good (OS 88% [71–95%], DFS 83% [65–92%]). HER2-positive tumors was the most pejorative group, with the worst prognosis for HER2+HR+ tumors (OS: 82% [59–93%], DFS: 65% [42–81%]) compared with HER2+HR− (OS: 87% [58%; 96%]; DFS: 82% [55–94%]).

### Multivariate analyses and prognostic factors

To assess which factors have independent impact on survival, multivariate analyses were performed for OS, DFS, LRFS, and MFS. All variables found significant at the 5% level in the univariate analysis, were included in the multivariate analysis. Cox proportional hazards models showed that overexpression of HER2 was a significant poor prognosis factor for OS, for DFS, for LRFS, and for MFS (Table 3).

**Table 3:** Multivariate analyses in 714 patients with pT1a-bN0M0 breast cancer.

	Variables	HR (95% CI)^1^	*P-*value
OS	HER2 status^2^	3.891 (1.583–9.565)	0.0031
	HR status^2^		0.3453
	SBR		0.2392
DFS	HER2 status^2^	3.571 (1.802–7.080)	0.0003
	SBR		0.2773
LRFS	HER2 status^2^	5.851 (1.551–22.077)	0.0091
MFS	HER2 status^2^	4.105 (1.886–8.936)	0.0004
	Age (>50 vs. ≤50)	3.420 (1.053–11.110)	0.0408
	SBR		0.0510

OS, overall survival; DFS, disease-free survival; LRFS, local recurrence-free survival; MFS, metastasis-free survival; HR, hormonal receptors; HER2, human epidermal growth factor receptor 2; SBR, Scarff Bloum and Richardson.

1 Hazard ratio (HR) and 95% confidence intervals (CI) are obtained from a multivariate Cox proportional regression model.

2 Positive versus negative.

For MFS, age was also significant in Cox model. Age >50 years was a bad prognostic factor for MFS.

## Discussion

Our analysis is one of the few studies reporting the 10-year outcomes of patients treated for small cancer breast in the southern France. The existing studies on patients with T1a-bN0M0 breast cancer treated with locoregional therapies reported 10-year DFS rates of more than 90%. Yet, a subset of these small early-stage breast cancers eventually relapses. Consistent with these data, our analysis showed that HER2 overexpression impacted significantly the prognosis and was associated with aggressive phenotype. On the basis of these results, many interrogations can be raised concerning the biology and the management of T1a-bN0M0 breast cancer.

In this analysis, the ratio of pT1a/T1b was ∼1/5, which is consistent with previous published data 2,13. Other publications have reported higher rates of T1a tumors, due to different selection criteria 14,15. This relative low rate of pT1a tumors could be explained partly by the difficulties to find sufficient materials for HER2 analysis. Literature varies concerning the prognosis of pT1a tumors compared with that of pT1b 2,13. We found no significant difference in survival and recurrence between pT1a and pT1b, possibly because of recruitment bias. Similar results were reported by Gonzales-Angulo et al. (DFS HR 1.59 [0.91–2.78], *P* = 0.103) 16. Tumor size was not a significant risk factor of recurrence or mortality. Hence, similar treatment strategies might be proposed for T1a and T1b, node-negative breast cancer.

It is interesting to note that multifocal SBCs, often linked to DCIS, were significantly correlated with HER2 amplification in the Italian study reported by Curigliano et al. 15. Consistently the majority of HER2-positive SBC in our analysis was ductal (90%), and was treated more often with mastectomy (18% vs. 3% HER2-negative; *P* < 0.001). These findings suggest that HER2-positive SBC with DCIS component might be at high risk of recurrence and that HER2 amplification may be involved in the transition from DCIS to invasive disease.

HER2 overexpression or gene amplification is relatively uncommon in SBC. It varies between 5% and 12% of all occurrences 2,16,17. Consistent with these data, HER2-positive tumors accounted for ∼6% of our cohort. HER2-positive tumors were mainly moderately or poorly differentiated tumors, as reported elsewhere 14,16. However, unlike other reports where younger age was found to be correlated with HER2 amplification, age was not a significant factor 14,16.

Our results confirmed that HER2 overexpression or amplification impacts significantly the prognosis of patients with T1a-bN0M0. An aggressive phenotype highly recurrent was observed in HER2-positive SBC. Press et al. were the first to publish in 1997 that HER2 amplification in the absence of adjuvant therapy is an independent predictor of poor prognosis, and is a stronger discriminant than tumor size 18. Pooled analysis of two studies: U.S. based single-center cohort of 965 patients 16 and an Italian study of 150 patients 14, demonstrated that HER2 positivity was significantly associated with a worse prognosis in terms of recurrence (HR 2.6, 95% CI 1.5–4.4, *P* < 0.001) 3. Several studies supported this finding 2,15,17,19,20.

In the HER2-positive cohort (*n* = 44 patients), patients HER2+HR+ showed the worse prognosis in our cohort at 10 years. A decreased benefit from hormone therapy was suggested in the HER2+ER+ subset of SBC 21,23. These patients are significantly more likely to relapse on ER antagonist (tamoxifen), resulting in a worse prognosis compared with HER2-negative group 14. Moreover, a benefit from aromatase inhibitors (i.e., letrozole) over tamoxifen was reported in HER2+ER+ 10,24,25. Taken together, these data highlight the role of a possible crosstalk between HER2 and HR, resulting in both intrinsic and acquired resistance to endocrine agents, and leading to poorer prognosis 26. Recently, Nahta and O'Regan suggested that a subset of HER2+ER+ breast cancers could be driven primarily by high level of ER expression, and may show a response more like HER2−ER+ breast cancers 27.

Five large clinical trials in patients with HER2-positive breast cancer demonstrated the benefit of trastuzumab, in combination with chemotherapy, in terms of survival and reduced relapse 7–9,28. However, there is no robust evidence for the use of trastuzumab in small node-negative breast cancer. Few results, from retrospective studies 29,30 and subgroup analysis 9, reported a benefit from adjuvant trastuzumab. A significant reduced risk of recurrence in SBC treated with local therapy was observed when adjuvant trastuzumab is combined to chemotherapy 9,29,30. Thus, the benefit of trastuzumab in T1a-bN0M0 HER2-positive breast cancers should not be ruled out.

Although generally well tolerated, adjuvant trastuzumab is associated with a significant increase in the incidence of cardiotoxicity, compared with chemotherapy alone 3. However, this cardiotoxicity was reversible and asymptomatic in most cases.

Thus during the decision–making process, the benefit should also be balanced against risks. To help weigh these benefits and risks, Kelly et al. assessed the number needed to treat (NNT) to save one patient from a breast event and the number needed to harm (NNH) by causing one adverse cardiac event using the meta-analysis performed by Dahabreh et al. 31. The NNH (ranging between 26 and 250) was larger than NNT (13–34) for patients treated with trastuzumab.

Consistently, French retrospective study (AERIO) had reported the outcomes of 97 patients with T1a-bN0 breast cancer. After a median follow-up of 29 months, a nonsignificant benefit in terms of recurrence was found for trastuzumab-based therapy 32. They estimated at 11 the NNT to prevent one breast cancer-related event. At 41-month follow-up, updated NNT was at seven 33.

In the light of our results and considering the low risk, adjuvant trastuzumab might be proposed particularly for patients with HER2+HR+ SBC, who are likely to display resistance to hormone therapy.

A phase-2 single-arm study (NCT00542451) is currently investigating the weekly administration of trastuzumab plus paclitaxel, in women with T1, node-negative, HER2-positive tumors. The awaiting results will provide supportive evidence for the management of SBC. Different pattern for trastuzumab-based regimen could be also proposed for investigation 34.

In conclusion, with the introduction of screening programs for breast cancer, it appears necessary to identify molecular predictors of prognosis within pT1a-bN0 SBC subtypes. The genomic signature could be used to identify a low-risk HER2-positive subgroup of patients with a favorable outcome, thus avoiding overtreatment and suggesting less intensive treatment strategies 35,36. Although uncommon, our data underlined the poor prognosis of HER2+HR+ tumors treated with hormone therapy. A personalized management through risk-stratified recommendations is needed in order to adjust the adjuvant treatment according to the molecular type of the tumor. Considering the retrospective nature of this study and the absence of a control arm, randomized studies are required. However, conducting such trials appears very tricky, assuming the need of a large number of patients and long follow-up to show rare breast cancer-related events in SBC.

## Conflict of Interest

None declared.
